# A Race Against Time: Contralateral Acute Limb Ischemia During Catheter‐Directed Thrombolysis for Peripheral In‐Stent Thrombosis—A Case Report

**DOI:** 10.1155/cric/8253871

**Published:** 2026-11-04

**Authors:** Vineet Madishetty, Abhinav Singh, Badal Sojitra, Alexander Yaylayan, Kevin Schneider, Merrill Krolick

**Affiliations:** ^1^ Internal Medicine/Cardiology HCA Healthcare/USF Morsani College of Medicine GME/HCA Florida Largo Hospital Largo Florida USA

**Keywords:** acute limb ischemia, catheter-directed thrombolysis, contralateral limb ischemia, endovascular therapy, in-stent thrombosis, limb salvage, mechanical thrombectomy, peripheral arterial disease

## Abstract

**Background:**

Acute limb ischemia (ALI) from peripheral in‐stent thrombosis is a vascular emergency requiring urgent revascularization. Catheter‐directed thrombolysis (CDT) is an established treatment option in selected patients, but development of contralateral ALI during active thrombolysis is rarely described.

**Case Summary:**

A 70‐year‐old man with severe peripheral arterial disease, prior bilateral lower extremity stenting, coronary artery disease, diabetes mellitus, chronic kidney disease, and active tobacco use presented with acute left lower extremity pain, coolness, and absent pulses with preserved motor function most consistent with Rutherford Class IIa ALI. Imaging demonstrated extensive thrombosis involving previously placed left iliac and femoropopliteal stents. The patient underwent mechanical aspiration thrombectomy, balloon angioplasty, and left‐sided CDT. During ongoing thrombolytic therapy, he developed new right lower extremity discoloration and absent pulses with preserved motor and sensory function—also most consistent with Rutherford Class IIa ALI. Repeat angiography demonstrated restored left‐sided perfusion but new occlusion involving the right common femoral and superficial femoral arterial stents. Mechanical aspiration thrombectomy followed by right‐sided CDT restored bilateral limb perfusion.

**Discussion:**

Contralateral ALI developing during CDT may have multifactorial causes, including underlying thrombotic susceptibility as well as local access or sheath‐related thrombosis. This case highlights the importance of bilateral vascular surveillance during thrombolytic therapy and prompt evaluation of new ischemic findings.

Take‐Home Message

During catheter‐directed thrombolysis (CDT), new contralateral ischemic findings should prompt immediate bilateral vascular reassessment, evaluation for both systemic and access‐related causes, and timely revascularization when indicated.

## 1. Background

Peripheral arterial disease (PAD) is a manifestation of systemic atherosclerosis associated with substantial morbidity, particularly among patients with diabetes, chronic kidney disease, coronary artery disease, and tobacco use [1]. Progressive or acute thrombotic occlusion of diseased peripheral arteries may result in acute limb ischemia (ALI), a vascular emergency characterized by an abrupt reduction in limb perfusion that can lead to tissue loss, amputation, and death without timely recognition and revascularization [1, 2].

Endovascular treatment has become an important component of contemporary management of complex PAD and ALI, with available strategies including percutaneous transluminal angioplasty, stent‐based intervention, mechanical or aspiration thrombectomy, and CDT [2, 3]. Although these approaches can restore perfusion and avoid open surgery in appropriately selected patients, peripheral in‐stent thrombosis remains an important cause of ALI, particularly in patients with extensive prior aortoiliac or femoropopliteal intervention [3, 4]. During CDT, close clinical surveillance is required because limb viability may change rapidly and complications such as bleeding, distal embolization, progression of ischemia, and compartment syndrome may occur [5].

Contralateral ALI developing during active thrombolytic therapy is rarely described. When new ischemia develops in the access limb, both systemic thrombotic susceptibility and local procedural mechanisms, including sheath‐related flow limitation, vessel injury, plaque disruption, or access site thrombosis should be considered. We report a 70‐year‐old male with advanced PAD and multiple prior lower extremity interventions who presented with left lower extremity ALI due to extensive peripheral in‐stent thrombosis and subsequently developed acute contralateral right lower extremity ischemia during ongoing CDT. This case highlights the importance of bilateral vascular surveillance during thrombolytic therapy and prompt evaluation of new ischemic findings in both the treated and access limbs.

## 2. Case Presentation

A 70‐year‐old male with severe peripheral artery disease status post multiple bilateral lower extremity interventions presented with a one‐day history of severe left lower extremity pain, coolness, and absent pulses. His medical history was notable for coronary artery disease status post coronary artery bypass grafting (CABG) and prior percutaneous coronary intervention (PCI), hypertension, hyperlipidemia, chronic kidney disease, diabetes mellitus, active tobacco use, and extensive PAD requiring prior bilateral lower extremity stenting. His vascular history was also significant for prior left lower extremity compartment syndrome requiring fasciotomy and a right superficial femoral artery (SFA) thrombectomy, angioplasty, and stenting approximately 4 months prior. His preadmission antithrombotic regimen included clopidogrel without aspirin; however, medication adherence could not be reliably confirmed.

On presentation, the patient′s left lower extremity was painful, cool, and pulseless, with preserved motor function. Based on the available clinical findings, the presentation was most consistent with Rutherford Class IIa ALI. Computed tomography angiography demonstrated extensive thrombosis involving the previously stented left iliac segment with extension through the superficial femoral and popliteal arteries. Intravenous unfractionated heparin was initiated, and the patient was taken emergently to the catheterization lab for peripheral angiography and attempted limb salvage.

Peripheral angiography demonstrated extensive thrombotic occlusion involving the left iliac and femoropopliteal arterial segments, including previously implanted stents. Right common femoral access was obtained under ultrasound guidance and a 7F × 45 cm crossover sheath was advanced into the left arterial system. Mechanical aspiration thrombectomy was performed within the thrombosed left iliac and femoropopliteal stents. Because substantial residual thrombus and impaired distal flow persisted, balloon angioplasty was performed using a 6 × 100 mm balloon. A tPa infusion catheter was then positioned across the residual thrombotic segment, and CDT was initiated with alteplase at 1 mg/h in addition to continuous intravenous unfractionated heparin (Figure 1). The patient was transferred to the intensive care unit for close monitoring during thrombolytic therapy. Serial examinations included assessment of limb perfusion, motor, and sensory function. In the absence of clinical deterioration, repeat angiography was initially planned to reassess residual thrombus burden and distal perfusion.

**Figure 1:**
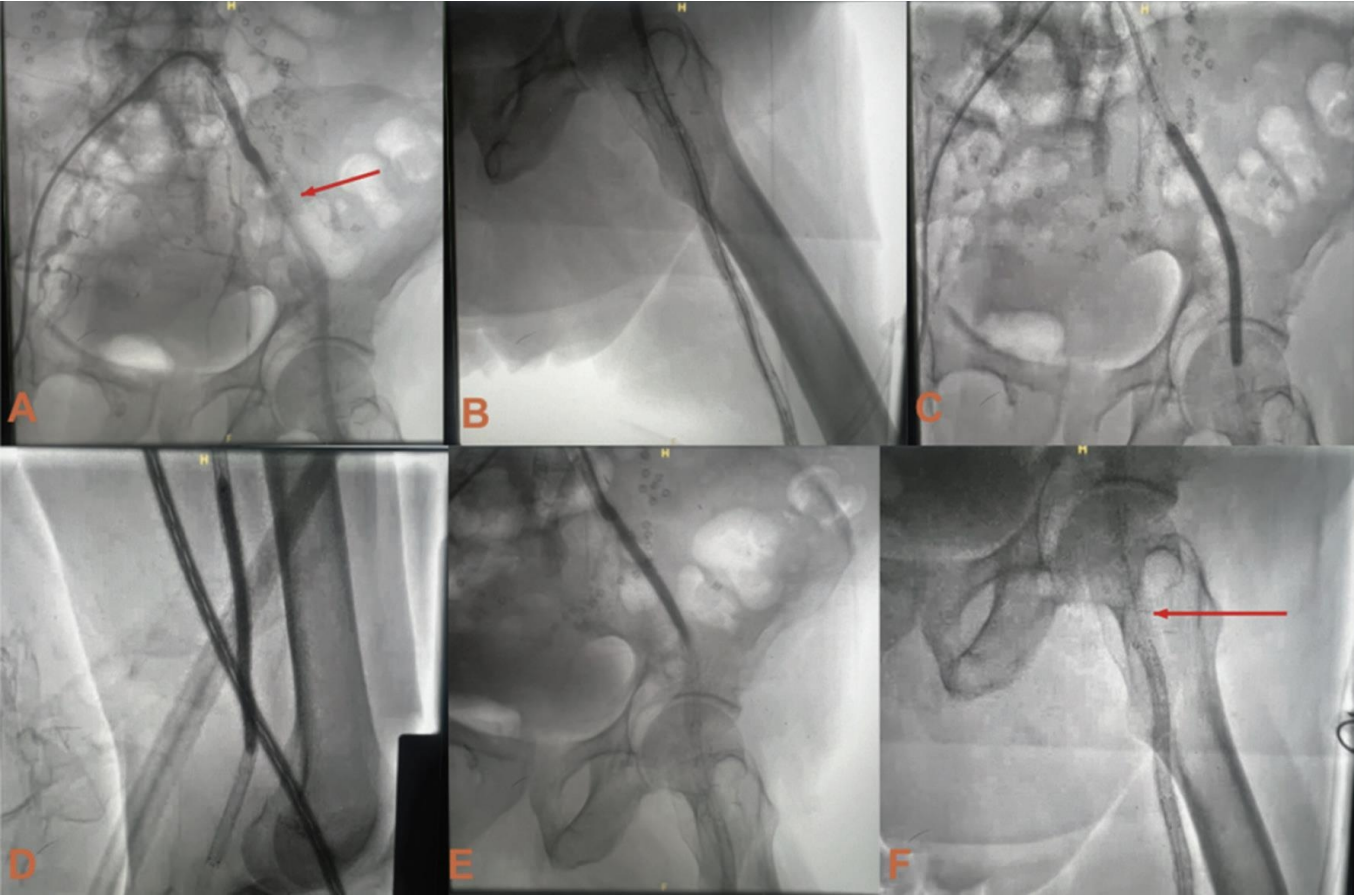
Initial left lower‐extremity angiography and endovascular treatment. (A) Angiography demonstrating thrombotic occlusion of the left iliac arterial segment with markedly reduced distal perfusion (arrow). (B) Previously implanted stents extending from the left external iliac artery into the common femoral arterial segment. (C‐D) Mechanical aspiration thrombectomy followed by balloon angioplasty of the thrombosed left iliac and superficial femoral arterial stents. (E) Improved antegrade flow following aspiration thrombectomy and balloon angioplasty, with residual thrombus prompting adjunctive catheter‐directed thrombolysis. (F) Thrombolysis catheter positioned across the residual thrombotic segment for continuous alteplase infusion (arrow).

Within hours of initiation of left‐sided CDT, the patient developed new right lower extremity discoloration and absent pulses. Motor and sensory function remained preserved. STAT arterial duplex ultrasound demonstrated occlusion of the right common femoral (CFA) and superficial femoral arterial (SFA) stents with minimal distal flow. Based on the available clinical findings, the right‐sided event was most consistent with Rutherford Class IIa ALI. Because arterial Doppler signal status was not documented in the available record, definitive distinction between Rutherford Classes I and IIa ischemia was limited.

Repeat peripheral angiography was performed after approximately 27 hours of left‐sided thrombolytic infusion. Angiography demonstrated restoration of left lower‐extremity perfusion with patent previously treated stents and two‐vessel distal runoff (Figure 2). However, a new complete thrombotic occlusion of the right common femoral artery with extension into the previously implanted SFA stents was identified. Notably, the right common femoral artery had served as the access site for the initial left‐sided intervention. Therefore, an access‐ or sheath‐related contribution to the subsequent right‐sided thrombosis could not be excluded on the basis of the available procedural information. The initial right femoral sheath was removed, and hemostasis was achieved. New left common femoral arterial access was then obtained, and a 7F sheath was advanced into the right common iliac artery. Mechanical aspiration thrombectomy of the right common femoral and superficial femoral arterial segments was performed. Although thrombus burden was reduced, substantial residual thrombus remained with impaired flow beyond the popliteal artery. Because substantial residual thrombus and inadequate distal perfusion persisted after aspiration thrombectomy, an alteplase bolus was administered, and a thrombolysis catheter was positioned within the right lower extremity arterial system (Figure 3). Catheter‐directed alteplase was continued at 1 mg/h in conjunction with intravenous unfractionated heparin.

**Figure 2:**
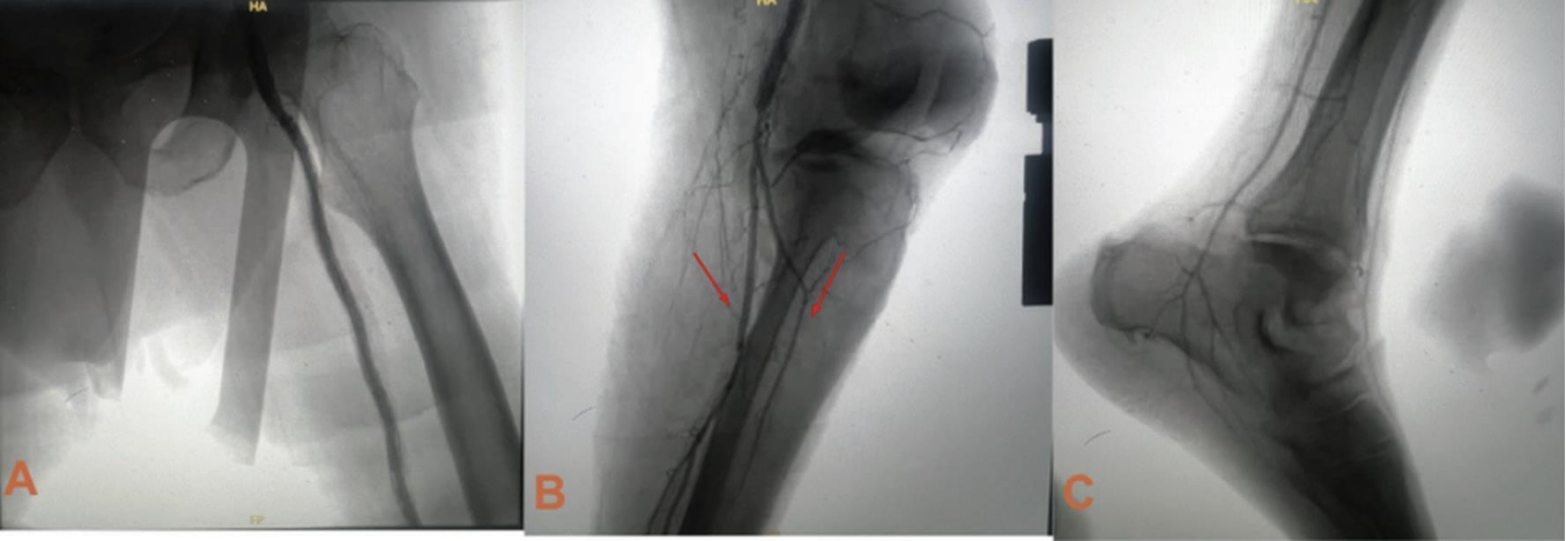
Restoration of left lower extremity perfusion following catheter‐directed thrombolysis. (A–C) Repeat angiography after approximately 27 h of alteplase infusion demonstrating restoration of antegrade flow through the previously thrombosed left lower extremity arterial segments and two‐vessel distal runoff (arrows). The previously treated iliac and femoropopliteal stents remained patent.

**Figure 3:**
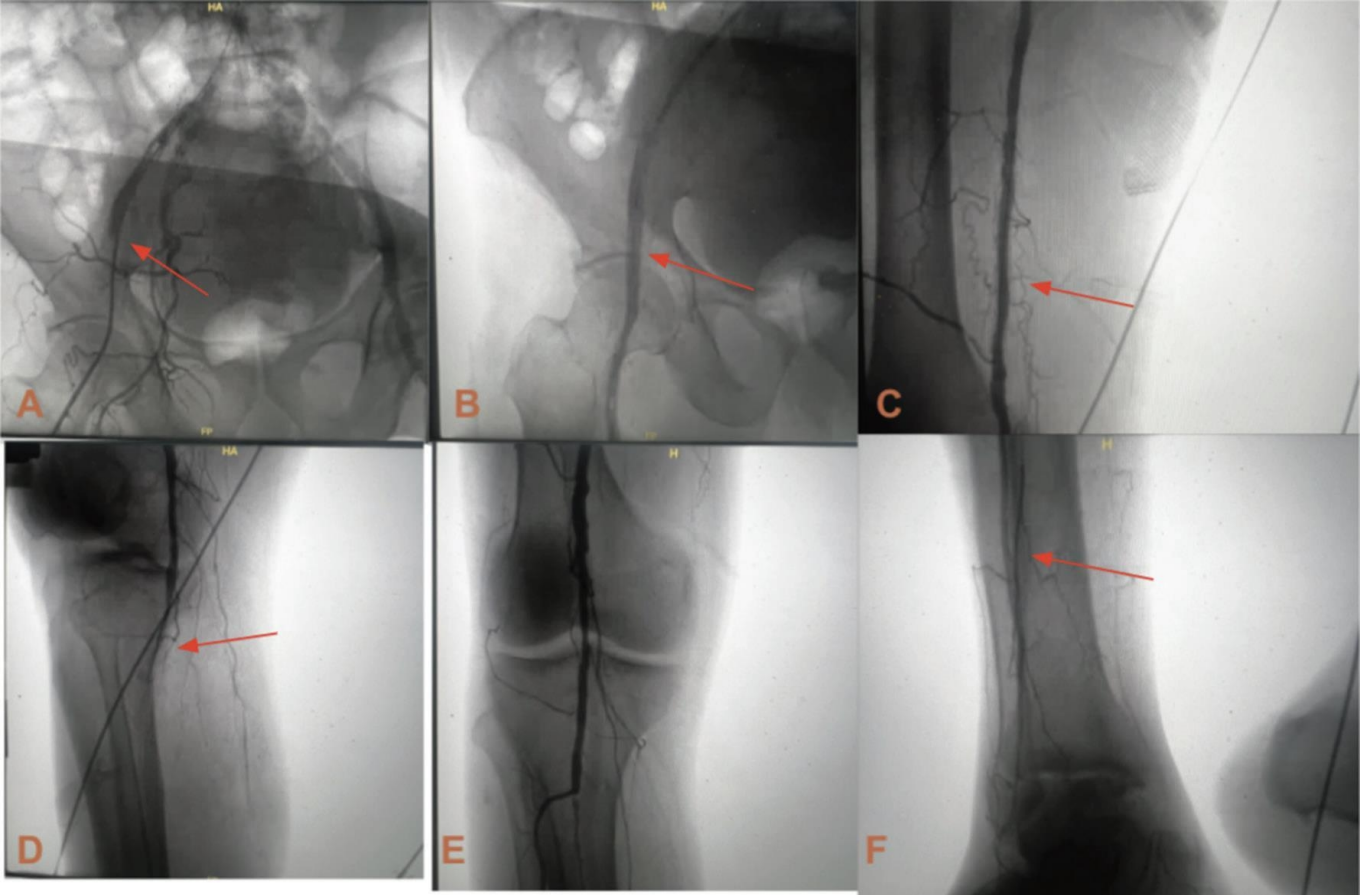
Contralateral right lower‐extremity acute limb ischemia and subsequent endovascular treatment. (A) Repeat angiography demonstrating new thrombotic occlusion of the right common femoral artery extending into the previously implanted superficial femoral artery stents (arrow). (B,C) Restoration of flow through the right common femoral and superficial arterial segments following mechanical aspiration thrombectomy. (D,E) Persistent thrombus burden with impaired flow distal to the right popliteal artery (arrows). (F) Thrombolysis catheter positioned within the right lower‐extremity arterial system for continued alteplase infusion (arrow).

After approximately 48 h of right‐sided CDT, repeat angiography demonstrated restoration of distal perfusion with widely patent previously implanted right common femoral arterial stents and single‐vessel runoff through the anterior tibial artery (Figure 4). The previously treated left iliac system remained widely patent without significant residual thrombus.

**Figure 4:**
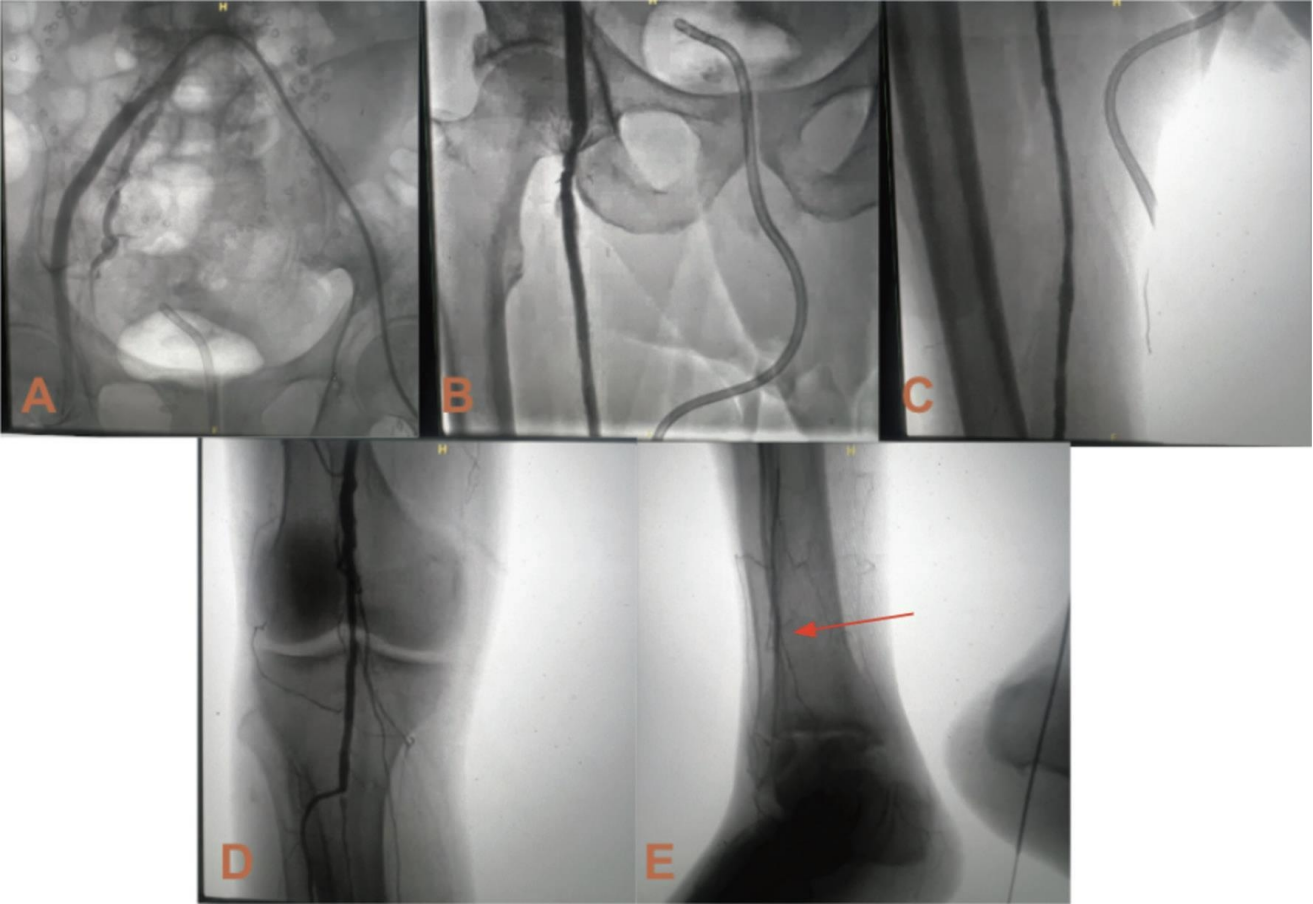
Final right lower extremity angiography following catheter‐directed thrombolysis. (A–E) Final angiography demonstrating restoration of right lower extremity perfusion with widely patent previously implanted stents within the distal common femoral arterial segment and single‐vessel distal runoff through the anterior tibial artery (arrow).

The subsequent hospital course was complicated by left lower extremity cellulitis with methicillin‐sensitive *Staphylococcus aureus*. Blood cultures were negative and there was no clinical or imaging evidence concerning for peripheral stent infection. The cellulitis was treated medically with appropriate antibiotic therapy and the patient was then discharged in stable condition. Given recurrent peripheral stent thrombosis despite reported compliance with clopidogrel therapy prior to index hospitalization, the patient was initiated on an individualized antithrombotic regimen of rivaroxaban 20 mg once daily plus ticagrelor 90 mg twice daily, withholding aspirin. Although inadequate clopidogrel effect was considered clinically, medication adherence before admission was uncertain, and platelet‐function or pharmacogenetic testing was not documented in the available record; thus, clopidogrel nonresponse could not be reliably confirmed. The use of full‐dose rivaroxaban represented an individualized treatment decision in the setting of recurrent arterial stent thrombosis rather than a guideline standard PAD regimen.

## 3. Discussion

This case highlights the complexity of managing ALI in a patient with diffuse, heavily instrumented peripheral arterial disease and multiple overlapping thrombotic risk factors. Peripheral in‐stent thrombosis is a recognized complication following lower extremity endovascular intervention and may result in ALI requiring urgent revascularization [4]. Contralateral ischemic symptoms have previously been described during longitudinal follow‐up after unilateral peripheral intervention; however, acute contralateral ALI developing during ongoing CDT is less well characterized [6]. Importantly, the right common femoral artery in this case had served as the access site for the initial left‐sided intervention. Therefore, the mechanism of the subsequent right‐sided thrombosis cannot be attributed definitively to a systemic prothrombotic state. Both underlying thrombotic susceptibility and local procedural mechanisms, including sheath‐related flow limitation, plaque disruption, vessel injury, or local thrombosis, must be appropriately considered.

Contemporary guidelines for management of ALI recommend close clinical reassessment because limb viability may change rapidly during treatment [7]. In this case, new right lower extremity discoloration and loss of pulses developed during left‐sided CDT, whereas motor and sensory function remained preserved. Urgent vascular imaging demonstrated new occlusion involving the right CFA and SFA stents. These findings emphasize the importance of bilateral rather than exclusively ipsilateral vascular surveillance during thrombolytic therapy. New ischemic findings in the access limb should additionally prompt evaluation for access or sheath‐related complications.

This case also illustrates the complementary roles of mechanical aspiration thrombectomy and CDT in complex ALI. Endovascular strategies including thrombectomy, angioplasty, and CDT may be used individually or in combination according to lesion anatomy, thrombus burden, ischemic severity, and response to initial therapy [8]. In the present case, initial treatment of the left lower extremity included mechanical aspiration thrombectomy followed by balloon angioplasty. Because substantial residual thrombus and impaired distal perfusion persisted, adjunctive CDT was performed. A similar multimodal strategy was subsequently required for the contralateral limb.

The role of pharmacomechanical and rheolytic thrombectomy in ALI continues to evolve. In a recent systematic review and meta‐analysis, Ciofani et al. found no significant differences between pharmacomechanical thrombectomy and CDT in technical success, 30‐day limb loss or mortality, or 12‐month primary patency, limb salvage or survival [9]. Pharmacomechanical thrombectomy was associated with shorter hospitalization but higher rates of distal embolization and acute kidney injury [9]. These findings suggest that neither strategy is universally preferable and that treatment selection should account for the need for rapid reperfusion, thrombus burden, anatomy, renal function, bleeding risk, and procedural expertise. Rheolytic thrombectomy represents one pharmacomechanical option for rapid thrombus debulking in selected patients. More broadly, recent comparative evidence evaluating pharmacomechanical thrombectomy against CDT has not demonstrated clear overall superiority of either strategy [9]. Although an endovascular‐first strategy was successful in this patient, open and hybrid revascularization remain important alternatives in selected patients with ALI [7]. Surgical thrombectomy, bypass surgery, endovascular intervention, and hybrid approaches may all have roles depending on limb viability, anatomy, thrombus burden, comorbidity, and available expertise [7].

Contemporary multicenter data from the ALIVE Registry further demonstrate that endovascular, open, and hybrid approaches all remain part of current ALI practice [10]. A recent case report and proposed management algorithm by Antonopoulos et al. additionally illustrates the potential role of hybrid revascularization in selected complex ALI presentations [11].

The antithrombotic strategy selected at discharge also warrants careful consideration. Following lower extremity revascularization for PAD, VOYAGER‐PAD demonstrated that rivaroxaban 2.5 mg twice daily in combination with aspirin reduced the composite risk of major cardiovascular and limb events [12]. The 2024 ACC/AHA PAD guideline subsequently recommends low‐dose rivaroxaban 2.5 mg twice daily combined with low‐dose aspirin following lower extremity revascularization to reduce major adverse cardiovascular and limb events [13]. In this case, however, the treating team selected an individualized regimen of rivaroxaban 20 mg once daily plus ticagrelor 90 mg twice daily, with aspirin withheld, in the setting of recurrent peripheral stent thrombosis. Given the absence of well‐defined evidence for this specific clinical scenario, antithrombotic therapy was individualized based on the patient′s recurrent thrombotic events and perceived ischemic risk. This highlights an area of uncertainty, as the optimal intensity and composition of antithrombotic therapy for patients with recurrent peripheral in‐stent thrombosis remains poorly defined and often requires a case‐by‐case approach.

The possibility of inadequate clopidogrel effect also requires cautious interpretation. Although the patient reportedly had been receiving clopidogrel before admission, medication adherence could not be reliably confirmed, and platelet‐function or pharmacogenetic testing was not documented in the available medical record. Therefore, clopidogrel nonresponse remained a clinical consideration rather than a confirmed mechanism. In EUCLID, ticagrelor was noted to be not superior to clopidogrel for reduction of cardiovascular events in patients with symptomatic PAD [14]. The optimal intensity and composition of antithrombotic therapy after recurrent peripheral arterial stent thrombosis remain uncertain and require individualized balancing of ischemic and bleeding risk.

## 4. Conclusion

This case describes contralateral ALI developing during CDT for unilateral peripheral in‐stent thrombosis. The event highlights the importance of bilateral vascular surveillance during thrombolytic therapy and the need to promptly evaluate new ischemic findings in both the treated and access site limbs. In this setting, both underlying thrombotic susceptibility and local access or sheath‐related mechanisms should be considered. Multimodal revascularization using mechanical aspiration thrombectomy, balloon angioplasty, and CDT successfully restored bilateral limb perfusion. The case also underscores the uncertainty surrounding optimal antithrombotic therapy in patients with recurrent peripheral stent thrombosis.

**Nomenclature**
ALI: acute limb ischemia
CABG: coronary artery bypass grafting
CAD: coronary artery disease
CDT: catheter‐directed thrombolysis
CKD: chronic kidney disease
CTA: computed tomography angiography
MSSA: methicillin‐sensitive *Staphylococcus aureus*
PAD: peripheral arterial disease
PCI: percutaneous coronary intervention
PTA: percutaneous transluminal angioplasty
SFA: superficial femoral artery

## Funding

This research was supported (in whole or in part) by HCA Healthcare and/or an HCA Healthcare–affiliated entity. The views expressed in this publication represent those of the authors and do not necessarily represent the official views of HCA Healthcare or any of its affiliated entities.

## Ethics Statement

Written informed consent was obtained from the patient for publication of this case report and any accompanying images. All identifying information was removed to protect patient confidentiality in accordance with HIPAA standards. Formal IRB approval was not required for this single‐patient case report under local institutional policy.

## Consent

Written informed consent was obtained from the patient for publication of this case.

## Conflicts of Interest

The authors declare no conflicts of interest.

## Data Availability

As a case report, the patient′s individual information is not available for review, in accordance with HIPAA compliance. Prior to development and submission of the manuscript, written informed consent was obtained from the patient.
